# Editorial: Prosthodontic research and clinical applications

**DOI:** 10.3389/fdmed.2026.1907894

**Published:** 2026-07-28

**Authors:** Artak Heboyan, Afsheen Maqsood, Naseer Ahmed

**Affiliations:** 1 Yerevan State Medical University, Yerevan, Armenia; 2 Bahria University, Islamabad, Pakistan; 3 Altamash Institute of Dental Medicine, Karachi, Pakistan

**Keywords:** biomaterials, CAD/CAM, digital dentistry, prosthodontics, restorative dentistry

Prosthodontics remains a vital branch of dentistry dedicated to the restoration and replacement of missing teeth and oral structures, with the ultimate goal of re-establishing oral function, esthetics, comfort, and quality of life. Contemporary prosthodontic and restorative practice involves a wide range of tooth-supported and implant-supported restorations, removable and fixed prostheses, resin-based materials, glass ionomer cements, ceramics, CAD/CAM materials, and emerging biomaterials. These options differ in their physical, chemical, biological, optical, and mechanical properties, as well as in their fabrication methods and long-term durability. Therefore, comparative evaluation of restorative materials, digital technologies, and clinical workflows remains essential for evidence-based decision-making.

This Research Topic was designed to provide an overview of current developments in prosthodontics, restorative dentistry, digital dentistry, prosthetic biomaterials, esthetic rehabilitation, prosthetic rehabilitation, implant-supported prostheses, precision prosthodontics, and clinical innovation. It also reflects the increasing role of nano- and computerized technologies, including CAD/CAM systems, digital scanning, finite element analysis, and artificial intelligence. As prosthodontics continues to evolve, clinicians and researchers must critically assess not only the immediate performance of new materials and technologies, but also their biological safety, clinical predictability, and long-term impact on dental, periodontal, and peri-implant tissues.

One important aspect of contemporary esthetic and restorative dentistry is the preservation of dental hard tissues. Although tooth bleaching is commonly performed to improve dental appearance, its effects on enamel should be carefully considered, particularly when future adhesive or prosthetic treatment is planned. In this context, Kuruba et al. showed that different bleaching protocols can affect enamel mineral content and surface structure. All agents caused some mineral loss and surface changes, with McInnes solution showing the greatest effect, while modified McInnes was less damaging and artificial saliva promoted partial remineralization. This highlights the need to consider enamel safety, not only whitening effectiveness.

Material interfaces represent another critical factor in the longevity of prosthodontic and restorative treatment. The sandwich technique remains clinically relevant because it combines the fluoride release and adhesion of glass ionomer cements with the esthetic and mechanical advantages of composite resins. However, the quality of the interface between these materials may influence restoration durability. Jouhar showed that bond strength in the sandwich technique depends on the combined effect of the glass ionomer type, composite material, and adhesive strategy. Resin-modified glass ionomer cement, nanohybrid composite, and etch-and-rinse adhesive provided the best immediate bonding performance, emphasizing the importance of selecting compatible restorative materials and protocols.

Digital dentistry has become increasingly important in prosthodontics, particularly in diagnosis, treatment planning, communication, and CAD/CAM fabrication. Intraoral scanners have improved many clinical workflows by reducing patient discomfort and enabling direct digital data acquisition. Nevertheless, their accuracy may be affected by clinical and anatomical factors, especially in complex preparations. Rokaya et al. showed that intraoral scanner accuracy for CAD/CAM post and core fabrication is influenced more by preparation geometry than scanner type. Longer post spaces reduced accuracy, while wider preparations improved it, emphasizing the technique-sensitive nature of digital scanning.

Together, the studies included in this Research Topic reflect the multidisciplinary and rapidly developing nature of prosthodontics and restorative dentistry. They connect biological preservation, biomaterial selection, adhesive optimization, and digital accuracy, all of which are essential for predictable oral rehabilitation. Whether clinicians are performing esthetic procedures, selecting restorative materials, designing layered restorations, or applying CAD/CAM workflows, treatment success depends on careful integration of material properties, tissue response, clinical technique, and patient-specific factors.

The broader significance of this Research Topic lies in its emphasis on evidence-based innovation. New restorative materials, prosthetic biomaterials, digital devices, and clinical techniques are continuously introduced into dentistry. However, their adoption should be guided by comparative research, laboratory validation, and clinical evidence. Particular attention should be given to the effects of prosthetic and restorative materials on enamel, dentin, periodontal tissues, peri-implant structures, and long-term rehabilitation outcomes.

Future research should move beyond isolated laboratory evaluation and adopt a more integrated, multidisciplinary approach to prosthodontic and restorative innovation. Further studies are needed to assess the long-term durability of restorative interfaces under aging, thermal, mechanical, and biological conditions; the effects of esthetic procedures on enamel and subsequent bonding; and the clinical accuracy of digital and CAD/CAM workflows in complex prosthodontic situations. Particular attention should also be given to the expanding role of artificial intelligence in prosthodontics. AI-based tools may support diagnostic imaging, implant planning, digital smile design, prosthetic design, biomaterial selection, peri-implant and periodontal monitoring, treatment outcome prediction, and patient-centered decision-making. However, their implementation must be supported by large-scale datasets, external validation, ethical data management, clinician oversight, and clear regulatory frameworks. AI should be considered as a technology that augments clinical judgment rather than replaces professional expertise.

In conclusion, this Research Topic contributes valuable evidence to the evolving fields of prosthodontics, restorative dentistry, and digital dentistry. By addressing enamel preservation, adhesive performance, biomaterial selection, and digital accuracy, the included studies support a more integrated, precise, and patient-centered approach to oral rehabilitation. The future of prosthodontics will depend not only on the introduction of new materials and technologies, but also on the ability to evaluate them critically and apply them responsibly in clinical practice.

